# Coccyx subluxation: Coccyx pain aggravated by the prone position

**DOI:** 10.1002/jgf2.570

**Published:** 2022-07-25

**Authors:** Yu Kumagai, Masahiro Biyajima, Ikuo Shimizu, Wataru Ishii

**Affiliations:** ^1^ Department of General Internal Medicine Nagano Red Cross Hospital Nagano Japan; ^2^ Center for Medical Education and Clinical Training Shinshu University School of Medicine Matsumoto Japan

**Keywords:** coccydynia, coccyx, postpartum, subluxation, tailbone

## Abstract

A 33‐year‐old woman presented with coccyx pain since her first vaginal delivery. On lateral plain radiographs, the tailbone was subluxated and dislocated ventrally.

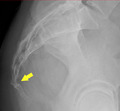

A 33‐year‐old woman presented with dyspepsia and coccyx pain. Since her first vaginal delivery, she had been experiencing discomfort during defecation followed by coccyx pain, which was persistent even after 18 months. Her condition gradually worsened, including difficulty in defecating and a sensation of residual stool. She had no obesity or adverse obstetric outcomes. Physical examination revealed coccyx pain during movements such as sitting and standing, and the pain was greatest when the patient was placed in a prone position. There was localized tenderness on the coccyx. On lateral plain radiographs, the coccyx was subluxated and displaced ventrally (Figure 1). Computed tomography also showed coccyx subluxation, but no other intra‐abdominal lesions were noted. Hence, analgesics and laxatives were prescribed.

**FIGURE 1:**
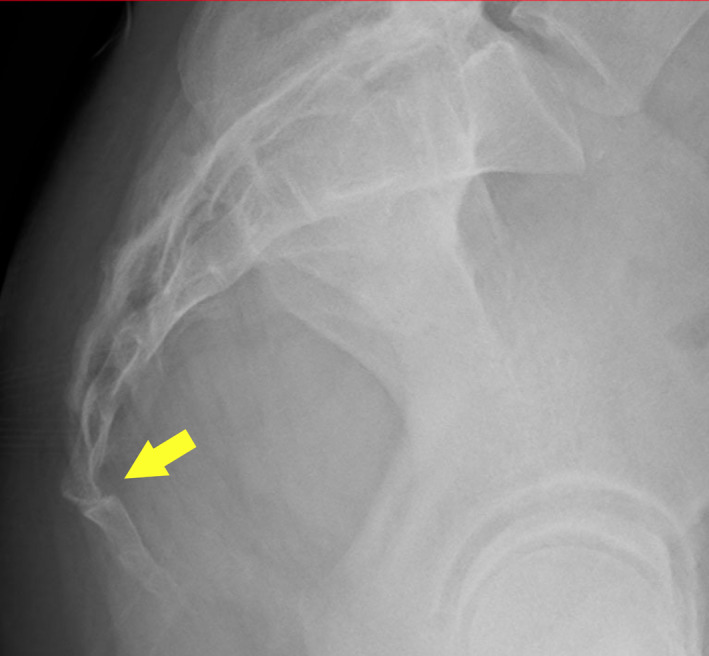
Roentgenogram of the sacrococcygeal vertebrae showing subluxation of the coccyx to the ventral side (*arrow*)

Postpartum coccydynia is a common symptom, which is often treated without proper evaluation of the patient's condition for traumatic injuries such as fracture, subluxation, and dislocation. Dystocia, obesity (body mass index ≥27), and more than two deliveries are associated with a high prevalence of tailbone dislocation.^1^ The treatment of traumatic tailbone dislocation remains controversial. Although normalizing the anatomical structure by manipulation of the dislocated fragment would seem plausible, manual repair by a transrectal approach could be highly invasive. Therefore, conservative treatment is considered, including the administration of analgesics, exercise therapy, local anesthesia, and avoiding painful positions. Caudal osteotomy is another treatment of choice for patients with chronic postpartum coccyx pain.^2^, ^3^

In our patient, the pain and difficulty in defecating were relieved by analgesics and laxatives; therefore, conservative treatment was successful. The pain that increased in the prone position was attributed to the subluxation of the tailbone to the ventral side. Coccyx pain results from abnormal mobility of the coccyx. There are many attachments to the coccyx and sacrococcygeal region. Stretching of the anterior sacrococcygeal ligament and traction of the pelvic parietal fascia in the prone position may cause coccyx pain.^4^ On the basis of expanded Postacchini and Massobrio classification, this case falls under type IV, which is often associated with coccydynia.^5^ Subluxation or dislocation of the coccyx during delivery usually results in the posterior dislocation as the fetal head pushes the coccyx backward. The reason for the anterior dislocation, in this case, may be that the tailbone, once dislocated posteriorly, gradually shifted ventrally because of daily activities performed after delivery such as sitting. Therefore, when women present with persistent pain in the buttock and a physical examination shows a localized tenderness in the coccyx, a subluxation of the coccyx related to vaginal delivery would be suspected. Furthermore, a history of worsening coccyx pain in a prone position may indicate anterior subluxation, rather than posterior. In such cases, in addition to the physical examination, a plain roentgenogram is useful for diagnosis.

## FUNDING INFORMATION

None declared.

## CONFLICT OF INTEREST

The authors have stated explicitly that there are no conflicts of interest in connection with this article.

## PARTICIPANT CONSENT

Written informed consent was obtained from the patient for publication of this case report and accompanying images.
